# Human Cell Assays for Synthesis-Dependent Strand Annealing and Crossing over During Double-Strand Break Repair

**DOI:** 10.1534/g3.116.037390

**Published:** 2017-02-07

**Authors:** Grzegorz Zapotoczny, Jeff Sekelsky

**Affiliations:** * Curriculum in Genetics and Molecular Biology, University of North Carolina at Chapel Hill, North Carolina 27599; † Department of Biology, University of North Carolina at Chapel Hill, North Carolina 27599; ‡ Integrative Program for Biological and Genome Sciences, University of North Carolina at Chapel Hill, North Carolina 27599

**Keywords:** double-strand break repair, crossing over, synthesis-dependent strand annealing

## Abstract

DNA double-strand breaks (DSBs) are one of the most deleterious types of lesions to the genome. Synthesis-dependent strand annealing (SDSA) is thought to be a major pathway of DSB repair, but direct tests of this model have only been conducted in budding yeast and *Drosophila*. To better understand this pathway, we developed an SDSA assay for use in human cells. Our results support the hypothesis that SDSA is an important DSB repair mechanism in human cells. We used siRNA knockdown to assess the roles of a number of helicases suggested to promote SDSA. None of the helicase knockdowns reduced SDSA, but knocking down BLM or RTEL1 increased SDSA. Molecular analysis of repair products suggests that these helicases may prevent long-tract repair synthesis. Since the major alternative to SDSA (repair involving a double-Holliday junction intermediate) can lead to crossovers, we also developed a fluorescent assay that detects crossovers generated during DSB repair. Together, these assays will be useful in investigating features and mechanisms of SDSA and crossover pathways in human cells.

Double-strand breaks (DSBs) are considered to be one of the most detrimental types of DNA damage. There are numerous mechanisms for repairing DSBs, broadly classified into end joining and homology-directed recombination (HDR). Among the latter, the double-strand break repair (DSBR) (Figure 1) model has been popular since it was proposed >30 yr ago (Szostak *et al.* 1983). A hallmark of this model is the double-Holliday junction (dHJ) intermediate, which has two of the four-stranded junctions originally hypothesized by Holliday (1964). In DSBR, as in Holliday’s model, specialized nucleases resolve Holliday junctions (HJs) by introducing symmetric nicks; independent resolution of the two HJs results in 50% of repair events having a reciprocal crossover. It has also been proposed that dHJs can be processed without the action of a nuclease if a helicase and topoisomerase migrate the two HJs toward one another and then decatenate the remaining link (Figure 1) (Thaler *et al.* 1987); this process has been called dissolution to distinguish it from endonucleolytic resolution (Wu and Hickson 2003).

**Figure 1:**
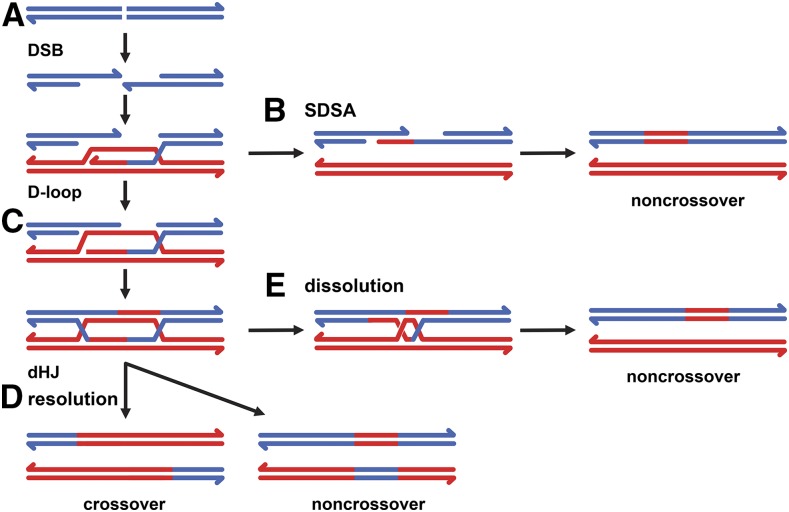
Models of DSB repair by homologous recombination. (A) Blue lines represent two strands of a DNA duplex that has experienced a DSB. HDR begins with resection to expose single-stranded DNA with 3′ ends (arrows). One of these can undergo strand invasion into a homologous duplex (red) to generate a D-loop; the 3′ invading end is then extended by synthesis. (B) In SDSA, the nascent strand is dissociated and anneals to the other resected end of the DSB. Completion of SDSA may result in noncrossover gene conversion (red patch, shown after repair of any mismatches). (C) An alternative to SDSA is annealing of the strand displaced by synthesis to the other resected end of the DSB. Additional synthesis can lead a dHJ intermediate. (D) In DSBR, the dHJ is resolved by cutting to generate either crossover or noncrossover products (one of two possible outcomes for each case is shown). (E) The dHJ can also be dissolved by a helicase-topoisomerase complex to generate noncrossover products.

In studies of DNA DSB repair resulting from transposable element excision in *Drosophila*, Nassif *et al.* (1994) noted that crossovers were infrequent and the two ends of a single DSB could use different repair templates. To explain these results, they proposed the synthesis-dependent strand annealing (SDSA) model (Figure 1). In addition to continued use of *Drosophila* gap-repair assays (*e.g.*, Kurkulos *et al.* 1994; Adams *et al.* 2003), other types of evidence have been interpreted as support for the SDSA model. In *Saccharomyces cerevisiae* meiotic recombination, gel-based separation and quantification of intermediates and products showed that noncrossovers are made before dHJs appear, suggesting that these noncrossovers are generated by SDSA (Allers and Lichten 2001). In vegetatively growing *S. cerevisiae*, Mitchel *et al.* (2010) studied repair of a small gap DSB in cells defective in mismatch repair. Based on the high frequency with which heteroduplex DNA tracts (regions that contain one template strand and one recipient strand) in noncrossover products were restricted to one side of the DSB, they concluded that most noncrossover repair occurred through SDSA. Miura *et al.* (2012) used an *S. cerevisiae* assay designed specifically to detect SDSA. A plasmid with a DSB was introduced into cells in which templates homologous to the two sides of the DSB were on different chromosomes, eliminating the possibility of a dHJ intermediate. Based on results of these various assays, many researchers now believe SDSA to be the most common mechanism of mitotic DSB repair by HDR (reviewed in Andersen and Sekelsky 2010; Verma and Greenberg 2016).

In mammalian cells, the direct-repeat GFP (DR-GFP) assay (Pierce *et al.* 1999) has been an instrumental tool for studying DSB repair by HDR. In this assay, an upstream *GFP* gene (*SceGFP*) is disrupted by insertion of an *I-Sce*I site, and a downstream *GFP* fragment (*iGFP*) serves as a template for repair. Gene conversion replaces the region surrounding the *I-Sce*I site in *SceGFP*, generating an intact *GFP* gene. This gene conversion has been suggested to arise through SDSA, but it is not possible to distinguish between SDSA and other noncrossover DSB repair in this assay (see Figure 1). Xu *et al.* (2012) developed a novel human cell assay in which gene conversion could be detected simultaneously at the DSB site and at another site ∼1 kbp away. They found that the two were often independent and concluded that SDSA is a major mechanism for DSB repair in human cells, but they also could not exclude DSBR as a possible source.

Development of the CRISPR/Cas9 system for genome engineering (Cong *et al.* 2013; Mali *et al.* 2013) provides additional emphasis on the importance of understanding SDSA mechanisms in human cells, as it has been suggested that replacement of multi-kilobase pair fragments after Cas9 cleavage, and probably other HDR events, occurs through SDSA (Byrne *et al.* 2015). We therefore designed an assay to detect DSB repair by SDSA in human cells. Here, we describe this assay and show that, as hypothesized, SDSA appears to be an important pathway for HDR in human cells. We report the effects of knocking down various proteins proposed to function during SDSA. We also describe a fluorescence-based assay for detecting crossovers generated during DSB repair. Use of these assays should help to further our understanding of DSB repair pathways used in human cells.

## Materials and Methods

### Construction of assay plasmids

The SDSA assay construct, pGZ-DSB-SDSA, was based on pEF1α-mCherry-C1 vector (catalog no. 631972; Clonetech). A fragment of *mCherry* was removed by cutting with *Nhe*I and *Hin*dIII and inserting annealed oligonucleotides containing an *I-Sce*I recognition sequence and a part of the *mCherry* sequence. The product, pEF1a-mCherry-I, had 350 bp of *mCherry* deleted and replaced with an *I-Sce*I recognition sequence. In parallel, 5′ and 3′ *mCherry* fragments, overlapping by 350 bp, were PCR-amplified and cloned into a vector containing a fragment of the *copia* retrotransposon from *D. melanogaster*. A fragment of *HPRT* was cloned out of the DR-GFP construct. This entire module (5′ *mCherry*–*copia*–3′ *mCherry*–*HPRT*) was PCR-amplified and cloned into the pEF1a-mCherry-I to produce pGZ-DSB-SDSA. The full sequence was deposited in GenBank under accession KY447299.

The crossover assay construct, pGZ-DSB-CO, was based on pHPRT-DRGFP (Pierce *et al.* 2001) and the intron-containing *mCherry* gene from pDN-D2irC6kwh (Nevozhay *et al.* 2013). The *iGFP* fragment was expanded to include the entire 3′ end of the transcribed region, and this was cloned into the *mCherry* intron of pDN-D2irC6kwh. This module (*mCherry* with an intron containing 3′ *GFP*) was cloned into the pHPRT-DRGFP vector cut with *Hin*dIII, so that it replaced the *iGFP* fragment and was in reverse orientation relative to the *SceGFP* gene. The full sequence of pGZ-DSB-CO was deposited in GenBank under accession KY447298.

### Generation of stably-transfected cell lines

U2OS and HeLa cells were cultured under normal conditions (DMEM + 10% FBS + pen/strep) for 24 hr until they reached 80% confluency before transfection with either SDSA or crossover assay constructs using a Nucleofector 2b Device (catalog no. AAB-1001; Lonza) and Cell Line Nucleofector Kit V (catalog no. VCA-1003; Lonza). At 1 wk post-transfection, appropriate antibiotics were added to select for the cells with a stably-integrated construct. pGZ-DSB-SDSA assay has a gene for neomycin resistance; cells receiving this construct were treated with 700 μg/ml G418 (catalog no. A1720; Sigma) for 1 wk and then single-cell clones were derived. pGZ-DSB-CO contains a *PGK1* gene that confers resistance to puromycin; cells receiving this construct were treated with 10 μg/ml puromycin (catalog no. P8833; Sigma) for 1 wk and then single-cell clones were derived. Initial attempts to determine copy number by Southern blot were unsuccessful; however, the analyses described below strongly suggested that the lines we characterized each carried a single insertion or possibly a single tandem array.

### DNA repair assays and flow cytometry

U2OS cells with pGZ-DSB-SDSA integrated were cultured in 10-cm dishes containing 10 ml of DMEM medium with high glucose (Corning) until split onto six-well plates at a concentration of 5 × 10^4^ cells/ml using 0.05% trypsin 0.53 mM EDTA solution (Corning). Upon reaching ∼60% confluency, the cells were treated with an siRNA reaction mixture (90 nmol siRNA and 8 μl lipofectamine 2000 reagent per well; Invitrogen). At 24 hr after transfection, the siRNA reaction mixture was replaced with the fresh culture medium. After 12 hr the cells were split so that knockdown could assessed in one half (see *qPCR evaluation of the siRNA knockdown efficiency*). The other half was treated with 100 μl *I-Sce*I–expressing adenovirus (Anglana and Bacchetti 1999) (previously titrated to a nonlethal concentration). After another 24 hr, the medium was replaced and thus the adenovirus removed. After another 72 hr, the cells were harvested and resuspended in 1× PBS (Corning) supplemented with 2% fetal bovine serum (FBS) and 5 mM EDTA, for flow cytometry acquisition on a BD LSRFortessa, using 488 and 561 nm lasers to detect the mCherry fluorescence.

U2OS cells with pGZ-DSB-CO integrated were cultured and treated under the same conditions. Flow cytometry acquisition was conducted on a BD FACSAriaII, using 388 and 532 nm lasers to detect GFP and mCherry fluorescence.

### U2OS genomic DNA isolation

Cells were cultured in a 15-cm dish until they reached 100% confluency, then rinsed with 1× PBS and harvested in 0.05% trypsin, 0.53 mM EDTA, by centrifuging for 3 min at 2000 rpm. Cells were washed with PBS and transferred to 1.5-ml microfuge tubes and spun for 10 sec to repellet. PBS was removed and cells were resuspended in TSM (10 mM Tris-HCl, pH 7.4; 140 mM NaCl; 1.5 mM MgCl_2_) with 0.5% NP-40 and incubated on ice for 2–3 min. After pelleting, cells were resuspended in 1 ml nuclei dropping buffer (0.075 M NaCl; 0.024 M EDTA, pH 8.0). The suspension was transferred to a 15-ml tube containing 4 ml nuclei dropping buffer with 1 mg Proteinase K (final Proteinase K concentration = 0.2 mg/ml), and 0.5% SDS. The cells were lysed overnight at 37°. The next day, an equal volume of phenol was added and mixed on an orbital shaker for 2 hr followed by a 5-min spin at 2000 rpm. The aqueous phase was transferred to a clean tube, an equal volume of chloroform was added, and the mix was incubated for 30 min on an orbital shaker. After spinning, the aqueous phase was transferred to a new tube and 0.1 vol 3 M NaOAc was added, followed by 1 vol isopropanol. The DNA was spooled out using a glass Pasteur pipette and resuspended overnight in 1 ml TE buffer (10 mM Tris-HCl, pH 8.0; 1 mM EDTA). The next day, the DNA was precipitated using 0.5 vol 7.5 M NH_4_OAc and 2 vol ethanol. DNA was spooled out and resuspended in 0.5 ml TE-4 buffer (10 mM Tris-HCl, pH 8.0; 0.1 mM EDTA). Samples were stored in 4° until analyzed.

### PCR analysis of the repair events

DNA from the BRCA2 knockdown repair events was isolated according to the protocol described in *U2OS genomic DNA isolation*, and used in a PCR reaction to amplify a desired DNA fragment for sequencing or fragment length characterization. A total of 1.5 μl DNA was added to each PCR mixture containing primer sets according to Supplemental Material, Table S1 in File S1, iProof polymerase (catalog no. 424264; BioRad) and buffer. PCR amplification reaction program was 33 cycles of the following: 20 sec at 98°, 20 sec at 64°, and 20–150 sec at 72°. Products were run on 1–1.5% agarose gels with ethidium bromide before being imaged.

### Western blot of BLM protein in siRNA-treated cells

Cells treated with siRNA as described in *DNA repair assays and flow cytometry* were harvested on the third day post-transfection using 0.05% Trypsin, 0.53% mM EDTA solution (Corning). After washing with 1× PBS, the cells were resuspended in a protein sample buffer (Tris-HCl; SDS; glycerol; bromophenol blue; 150 mM DTT) and boiled. A total of 20 μl of the protein sample was loaded on a 7.5% SDS-PAGE gel and the gel was run for 1–2 hr at 100 V. Protein was transferred to a PVDF membrane using a wet transfer method (1.5 hr at 90 V in 4°). The membrane was blocked in PBS with 5% powdered milk and incubated in PBS plus 0.1% Triton-X plus primary antibodies [rabbit anti-BLM (catalog no. 2179; Abcam) at 1:2000 and mouse anti-αTubulin (catalog no. T9026; Sigma) at 1:8000] overnight at 4° on a rocker. The membrane was then washed three times in PBS-T solution. HDRP-conjugated secondary antibodies were added (goat anti-rabbit at 1:5000 and goat anti-mouse at 1:100,000) and the blot was incubated for 1 hr at room temperature. The membrane was washed three times in PBS-T solution and then incubated in an ECL solution (Thermo Fisher Scientific) for chemiluminescence for 2 min. The Western blot image was taken using a BioRad Molecular Imager (ChemiDoc XRS+) or the X-ray film was developed using a developer.

### qPCR evaluation of the siRNA knockdown efficiency

Cells treated with siRNA as described in *DNA repair assays and flow cytometry* were harvested on the third day post-transfection using 0.05% Trypsin 0.53% mM EDTA solution (Corning). RNA was extracted using the manufacturer’s protocol for ReliaPrep RNA Cell Miniprep System (Promega). Purified RNA was used as a template to generate the cDNA library with QuantiTect Reverse Transcription Kit (catalog no. 205310; Qiagen). The qPCR mix contained gene-specific DNA primers, cDNA, and the QuantiTect SYBR Green PCR kit (catalog no. A 204141; Qiagen). Amplification and quantification was conducted on a RealTime PCR machine (QuantStudio 6 flex Real Time PCR System).

### Statistical analysis

Statistical comparisons were performed on the raw data (Tables S2 and S3 in File S1) using GraphPad Prism version 6.07 for Windows (GraphPad Software Inc., La Jolla, CA). In the case of BLM knockdown in the SDSA assay, one value (271% of control) was found to be a significant outlier based on the Grubb’s test using GraphPad QuickCalcs online (https://graphpad.com/quickcalcs/grubbs2/), and was excluded from further analysis.

### Data availability

All data are in the figures or the supplemental tables in File S1. Cell lines and plasmids are available on request. Sequences of assay constructs were deposited in GenBank under accession numbers KY447298 (pGZ-DSB-CO) and KY447299 (pGZ-DSB-SDSA).

## Results and Discussion

### An SDSA assay for human cells

**Figure 2:**
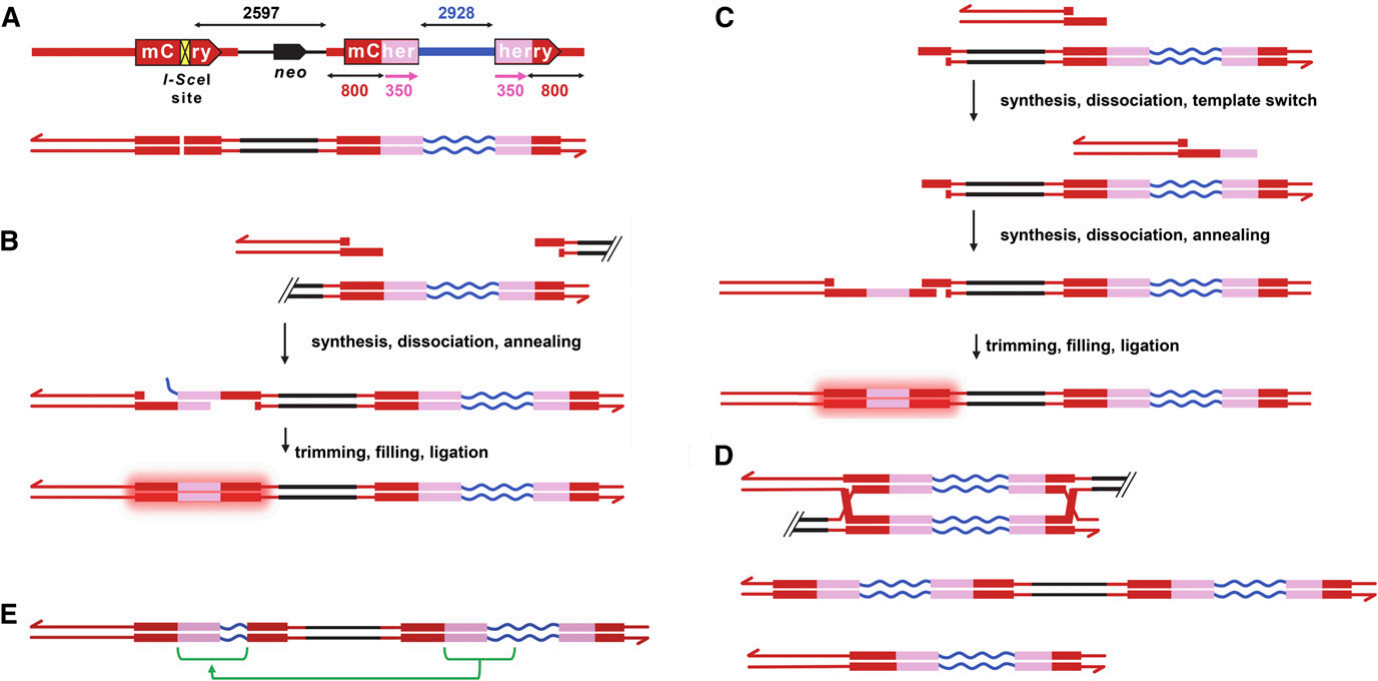
An SDSA assay for human cells. (A) Schematic of the assay construct. The *mCherry* coding sequence is represented with a large arrow, with the yellow box indicating the site at which a 350-bp fragment was removed and replaced with an *I-Sce*I site. The thick red lines designate the promoter and 5′ and 3′ untranslated regions. Downstream of *mCherry* is the *neo* selectable marker and vector sequences (black arrow and line, respectively). The repair template consists of 800 bp of homology to the left of the DSB site, then the 350-bp deleted fragment (magenta), a 3-kbp spacer (blue), another copy of the 350-bp fragment, and 800 bp of homology to the right end of the DSB site. The lines below are a representation of the same sequences as duplex DNA, for use in subsequent panels. Arrowheads indicate 3′ ends at the outer edges of the construct in all panels. (B) Two-ended SDSA repair. The DSB is shown as resected and paired with a template. This diagram shows interchromatid repair, where the template on the sister chromatid is used (whether or not it was cut by *I-Sce*I). Intrachromatid repair may also be possible; in this case, the black lines to the right of the DSB would be continuous with those to the left of the template. After strand exchange, synthesis into the 350-bp regions, and dissociation, complementary sequences can anneal. In the example shown here, the left end has been extended through the 350-bp fragment into the spacer; the right end synthesized only midway into the 350-bp fragment. After trimming of the spacer sequence, filling of gaps, and ligation, a restored *mCherry* is produced, resulting in red fluorescence. (C) One-ended sequential SDSA. This is similar to (B), except only the left end of the DSB has paired with the template. After synthesis and dissociation, the nascent strand can pair with the second half of the template. Additional synthesis can extend the nascent strand to provide complementarity to the other end of the DSB, allowing annealing and completion of SDSA. (D) If one or both ends invade the template and synthesis traverses the entire template, a dHJ can form (top). If the dHJ is dissolved or resolved in the noncrossover orientation, the product shown in the middle is generated. The bottom part of this panel shows the chromosomal product of crossover resolution (there is also an acentric extrachromosomal circle that will be lost). (E) A product produced by initiation of repair by SDSA but completion by end joining. In this case, part of the spacer has been copied into the upstream *mCherry* (sequences from the template are indicated with green lines).

To study SDSA in human cells, we used an approach conceptually similar to the *P*{*w^a^*} assay used in *Drosophila* (Adams *et al.* 2003; McVey *et al.* 2004). In this assay, if both ends of the DSB generated by *P* element excision are extended by synthesis from the sister chromatid, the nascent strands can anneal at repeats inside the *P*{*w^a^*} element, generating a product that is unique to SDSA and easily distinguishable by phenotype. To mimic this situation in human cells, we built a construct (Figure 2) that has an *mCherry* gene in which a 350-bp segment was replaced with the 18 bp *I-Sce*I recognition sequence, rendering the gene nonfunctional. When a DSB is generated by *I-Sce*I (Figure 2A), HDR can be completed using a downstream repair template. The repair template is split: each half has 800 bp of homology adjacent to the break site plus the 350 bp of deleted *mCherry* sequence. The two halves are separated by a 3-kbp spacer of unique sequence. Since the 350-bp sequence is on both sides of the spacer, it constitutes a direct repeat. We hypothesized that both ends of the *I-Sce*I–induced break will invade the side of the template to which they are homologous, either simultaneously or sequentially (Figure 2B). If synthesis on both sides extends through or far enough into the 350-bp repeat before the nascent strands are dissociated from the template, the overlapping regions can anneal to one another (Figure 2B). Completion of SDSA restores a functional *mCherry* gene at the upstream location.

The scenario above requires two-ended SDSA, but sequential one-ended SDSA is also possible (Figure 2C). If only one end of the break invades the downstream template, is extended by repair synthesis, and is then dissociated from the template, the nascent strand will not be complementary to the other resected end; however, this nascent strand will have homology to the repeat on the other side of the template. A second cycle of strand exchange and repair synthesis using the other repeat could lead to addition of sequences complementary to the other resected DSB end. This would also restore a functional *mCherry* gene by SDSA.

A functional *mCherry* gene might also be generated through a combination of SDSA and DSBR. In the sequential SDSA scenario, the second strand exchange event could be processed into a dHJ. The product of dissolution or noncrossover resolution of such a dHJ will be identical to that of SDSA (Figure 2B), but if the dHJ is resolved as a crossover, generation of a functional *mCherry* gene will be accompanied by a deletion of all sequences between the upstream *mCherry* and the downstream template. Dissolution or noncrossover dHJ resolution in this scenario cannot be distinguished from SDSA, but it should be noted that formation of such a dHJ intermediate still requires at least one cycle of D-loop disassembly—a key step that separates SDSA from DSBR (Figure 1).

Other types of repair that do not generate a functional *mCherry* are possible. A dHJ can be generated if synthesis extends through one *mCherry* 350-bp repeat, the entire spacer, and the other 350-bp repeat (Figure 2D). Processing of this dHJ would give a product in which the entire template, including the duplicated 350-bp sequences and the spacer, was copied into the upstream *mCherry* gene. Dissolution or noncrossover resolution would result in two copies of the template (Figure 2D, middle), whereas crossover resolution would delete intervening sequences (Figure 2D, bottom). Nonhomologous end joining (NHEJ) can restore or disrupt the *I-Sce*I recognition sequence, depending on whether it is precise or imprecise (not depicted). Hybrid repair, in which repair is initiated by HDR but completed by end joining instead of annealing, can give rise to an *mCherry* in which the 350-bp gap is not completely filled or, if synthesis extends into the spacer, in which part of the spacer is copied into the upstream *mCherry* gene (Figure 2E).

**Figure 3:**
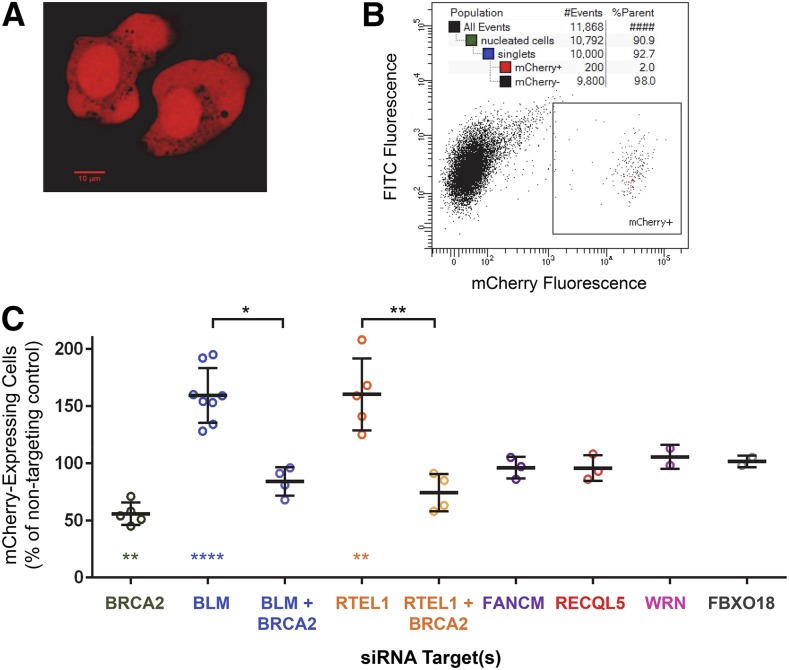
Results from the SDSA assay. (A) Cells fluorescing red due to mCherry expression after *I-Sce*I infection. (B) Flow cytometry of cells after *I-Sce*I expression. In this example, 10,000 singlet cells were assayed and 200 were gated as exhibiting red fluorescence. (C) Effects of siRNA knockdown on acquisition of mCherry expression (see *Materials and Methods*). Fluorescence frequencies from flow cytometry were normalized to NT; raw data are given in Table S2 in File S1. Error bars indicate SD. The ratio paired *t*-test was used to compare raw values for each siRNA target to its NT control and each double-knockdown to the single knockdown of the corresponding helicase. For helicase single knockdowns, *P* values were Bonferroni-corrected for multiple (six) comparisons. * *P* < 0.05, ** *P* < 0.01, **** *P* < 0.0001.

To generate cell lines with the SDSA repair construct, we transfected both U2OS and HeLa cells with linearized SDSA construct and used G418 to select stably-transfected lines. To induce DSBs, we infected cells with an adenovirus expressing *I-Sce*I (Anglana and Bacchetti 1999). We detected mCherry activity by fluorescence microscopy (Figure 3A and Figure S2A in File S1) as early as 2 d after viral infection. Stable expression persisted through months of cell culturing. Molecular analysis of genomic DNA from clones derived from single mCherry-positive cells confirmed the absence of the *I-Sce*I, restoration of a complete *mCherry* gene, and the presence of an intact repair template (Figure S1 in File S1). We quantified SDSA repair through flow cytometry (Figure 3B and Table S2 in File S1).

We obtained mCherry-positive cells in multiple isolates of each cell type, and we selected one U2OS cell isolate for further characterization. Since we intended to use siRNA to knock down proteins that might be required for SDSA, we first knocked down BRCA2 as a positive control. BRCA2 is essential for RAD51-mediated strand exchange and for initiation of HDR (Sharan *et al.* 1997). Consistent with this function, knocking down BRCA2 resulted in a significant decrease in red-fluorescing cells relative to the nontargeting (NT) siRNA-negative control (Figure 3C). Although there was substantial residual HDR, probably due to incomplete loss of BRCA2 (Figure S3 in File S1), we conclude that SDSA does occur in human cells and that our assay can be used to study this process.

### An assay for detecting crossovers generated during DSB repair in human cells

**Figure 4:**
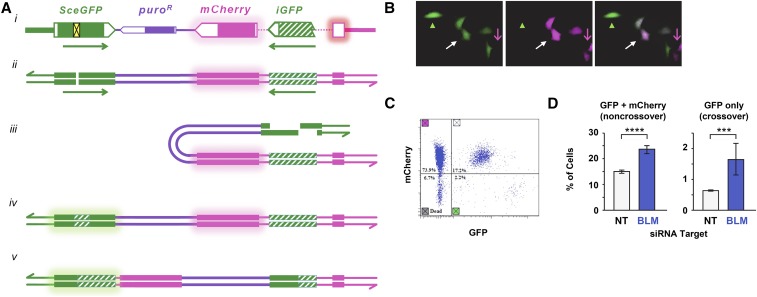
An assay to detect crossovers generated during DSB repair. (A) Schematic of the assay. The diagram at the top (i) has the *SceGFP* gene in solid green, with the yellow box indicating insertion of an *I-Sce*I site. The modified *iGFP* fragment (with the entire 3′ end of the gene) is indicated in hatched green. The *mCherry* gene is shown in magenta, with the intron represented as a dotted line. (ii) Representation of the construct as double-stranded DNA. (iii) After DSB introduction and resection, resected ends can pair with the homologous *iGFP* template. The example here shows intrachromatid repair. (iv) Gene conversion without a crossover results in replacement of the *I-Sce*I site with *GFP* sequences, generating a functional *GFP* gene. mCherry expression is unaffected. (v) If a crossover is generated, the central region is inverted and mCherry expression is lost. Repair with the sister chromatid (interchromatid repair) may also be possible. In that case, a crossover results in a dicentric chromatid and an acentric chromatid; these products will not be recovered, but noncrossover gene conversion will be recovered. (B) A field of cells after *I-Sce*I expression, showing the green (left) and red (middle, magenta) channels, and a merged image (right). Green arrowhead marks a cell that gained GFP expression but lost mCherry expression (a crossover); white arrow indicates a cell that gained GFP expression and retained mCherry expression (noncrossover gene conversion); magenta arrow indicates a cell in which GFP was not converted. (C) Flow cytometry showing gating for GFP and mCherry fluorescence. (D) Increased noncrossover and crossover gene conversion after knocking down BLM. Bars indicate percentage of cells expressing GFP and mCherry (noncrossover gene conversion; upper right quadrant of flow cytometry output) or GFP only (crossovers; lower right quadrant). Error bars are SD based on three different wells per treatment. Raw data are in Table S3 in File S1. *** *P* = 0.0010, **** *P* < 0.0001 based on unpaired *t*-test.

D-loop dissociation is a critical step in the SDSA pathway. If the D-loop is not dissociated, 2nd-end capture may lead to formation of a dHJ intermediate (Figure 1C). It is thought that most dHJs formed in proliferating cells are processed by dissolution to give noncrossover products, but resolution by nicking can generate crossovers (Figure 1D). To complement our assay for SDSA, we developed an assay that detects crossovers generated after DSB formation (Figure 4A). Our assay is based on the DR-GFP gene conversion assay (Pierce *et al.* 2001), which has a *GFP* gene interrupted by an *I-Sce*I site (*SceGFP*), and a downstream *GFP* fragment (*iGFP*) that serves as a repair template. We added an *mCherry* gene with an intron and placed the *iGFP* fragment (modified to contain the entire 3′ end of *GFP*) inside the intron. This is in reverse orientation relative to *SceGFP*, to prevent the possibility of the single-strand annealing pathway. In cells transfected with this construct, mCherry is expressed but GFP is not. After DSB induction with *I-Sce*I, gene conversion of the *I-Sce*I site restores GFP expression. However, if gene conversion is accompanied by a crossover, the region between the *GFP* fragments becomes inverted, resulting in loss of mCherry expression.

We introduced this construct into both U2OS and HeLa cells and selected for stably-transfected lines that expressed mCherry. We tested several U2OS lines for response to *I-Sce*I introduction. We selected a line that consistently yielded cells that were positive for both GFP and mCherry (noncrossover gene conversions) and cells that were positive for GFP only (crossovers). In our standard protocol (see *Materials and Methods*), treatment of NT siRNA control cells with *I-Sce*I resulted in 15.6% of cells gaining GFP expression (Figure 4, B and C and Table S3 in File S1); 3.8% of these (0.6% of all cells) had lost mCherry expression. This is likely to be an underestimate of the crossover frequency because only intrachromatid crossovers can be recovered, as interchromatid crossovers result in dicentric and acentric products (see Figure 4 legend).

### Knocking down BLM or RTEL1 elevates SDSA frequency

In the model shown in Figure 1, SDSA diverges from dHJ pathways when a helicase dissociates the nascent strand from the template. Several helicases have been suggested to perform this step. *Drosophila* gap repair assays found roles for Blm and Fancm helicases in SDSA (Adams *et al.* 2003; Kuo *et al.* 2014). The *Arabidopsis* orthologs of these enzymes promote noncrossover recombination in meiosis, possibly by SDSA (Crismani *et al.* 2012; Séguéla-Arnaud *et al.* 2015). Sgs1, the yeast ortholog of Blm, is required for noncrossover recombination in budding yeast meiosis (De Muyt *et al.* 2012), and Sgs1 and Mph1 (the orthologs of Fancm) have been implicated in SDSA in vegetative cells (Mitchel *et al.* 2013). In *Schizosaccharomyces pombe*, Fml1, the ortholog of Fancm/Mph1, was suggested to promote SDSA in DSB repair during replication and in meiosis (Sun *et al.* 2008; Lorenz *et al.* 2012). Yet another helicase, RTEL-1, was hypothesized to disrupt D-loops in *Caenorhabditis elegans* meiosis (Barber *et al.* 2008; Youds *et al.* 2010). In human cells, RECQ5 is proposed to promote SDSA (Paliwal *et al.* 2014). Aside from the experiments in *Drosophila* and budding yeast, none of the assays performed could distinguish between SDSA and other pathways. Thus, we used our assay to ask whether the orthologs of any of these or related helicases affect SDSA in human cells.

We did not detect any change in the frequency of red-fluorescing cells after knocking down FANCM, RECQ5, WRN, or FBXO18 (Figure 3C and Table S2 in File S1). Knockdown of BLM or RTEL1 significantly altered the frequency of red-fluorescing cells; however, instead of decreasing SDSA as expected, both knockdowns resulted in an increase in red-fluorescing cells (Figure 3C). BLM has been shown to have several functions in HDR pathways, including in DSB end resection in a pathway redundant with *Exo*I (Zhu *et al.* 2008) and dHJ dissolution (Wu and Hickson 2003; Wu *et al.* 2006; Singh *et al.* 2008). Likewise, RTEL1, which was initially identified as a telomere length regulator and is responsible for T-loop disruption (Ding *et al.* 2004; Sarek *et al.* 2015), can also unwind DNA secondary structures and promote replication fork progression (Barber *et al.* 2008). To begin to assess how knocking down BLM and RTEL1 might increase SDSA, we knocked down each in combination with BRCA2. In simultaneous knockdowns, red-fluorescing cells were decreased from the frequency observed of BLM and RTEL1 single knockdowns (Figure 2C). The magnitude of the decrease was similar to that of the BRCA2 single knockdown relative to the negative control (44% decrease for BRCA2 relative to NT, 47% for BRCA2 + BLM relative to BLM alone, and 54% for BRCA2 + RTEL1 relative to RTEL1 alone), indicating that BLM and RTEL1 impact SDSA through functions after strand exchange into a homologous template.

We also tested the effects of knocking down BLM in the crossover assay. Transfection with siRNA to knockdown BLM resulted in 25% of cells gaining GFP expression (Figure 3C). This is a 60% increase compared to the NT control, similar to the average increase of 59% in the SDSA assay. Crossovers were elevated, with 6.4% of the GFP-positive cells (1.6% of all cells) having also lost mCherry expression. This is consistent with studies showing elevated spontaneous crossing over in cells from Bloom syndrome patients (German *et al.* 1977).

### Knocking down BLM or RTEL1 alters repair outcomes

To further develop our SDSA assay and gain additional insights into the effects of knocking down BLM and RTEL, we determined the structures of repair events produced in knockdown cells. We analyzed 55 clones derived from single red-fluorescing cells, including 23 from the NT control, 21 from BLM knockdown, 10 from FANCM knockdown, and one from RTEL1 knockdown. All but one had the structure expected of SDSA (Figure 2B). The remaining clone, which came from NT siRNA treatment, had lost *neo* and the template spacer, and therefore may have arisen from SDSA followed by DSBR with crossover resolution (Figure 1D). These results support our conclusion that cells with restored *mCherry* utilized SDSA to repair the DSB, perhaps occasionally coupled with use of DSBR.

**Figure 5:**
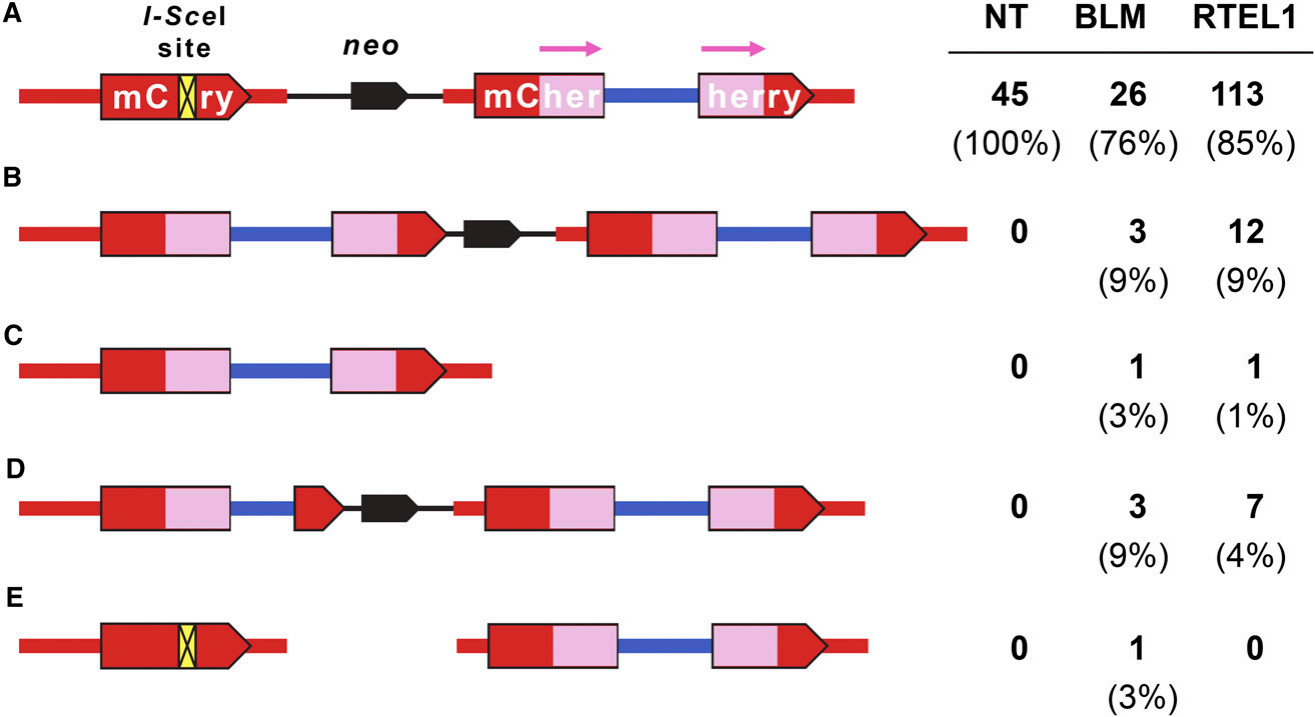
Structures of repair events in cells not expressing mCherry. Single cells not exhibiting red fluorescence were grown and then analyzed. (A) The starting construct, and the structure found in the majority of clones in each siRNA treatment group. (B) Copying of the entire template into the upstream *mCherry I-Sce*I site. This might occur through long-tract SDSA (one synthesis cycle or several) or a dHJ intermediate (as in Figure 2D, center). (C) Copying of the entire template with loss of intervening sequences. This is proposed to occur when a dHJ intermediate is resolved in the crossover orientation, as in Figure 2D, bottom. (D) Copying of part of the template into the upstream *mCherry* site, as predicted from initiation of SDSA but completion of repair by end joining, as in Figure 2E. (E) Retention of the *I-Sce*I site with loss of the *neo* and *ori* sequences.

We also analyzed cells that failed to produce mCherry. In the NT control, all 45 lines examined appeared to be identical to the initial construct (Figure 5A). We did not measure cleavage efficiency in our assay, but we titrated adenovirus infection to the highest dose that did not cause detectable cell lethality. Delivery of *I-Sce*I by adenovirus infection of HEK293 cells resulted in 85% of sites being cut (Anglana and Bacchetti 1999), so it is likely that most or all of the cells with intact *I-Sce*I sites are likely to result from cleavage followed by precise NHEJ using the 4 nt complementary overhangs left by *I-Sce*I.

The majority of clones from BLM or RTEL1 knockdown cells that did not produce mCherry also had an intact *I-Sce*I site; however, structures indicating other repair processes were observed in 11 out of 34 of these clones from BLM knockdown (*P* < 0.0001 compared to NT) and 24 out of 133 of these clones from RTEL1 knockdown cells (*P* = 0.0007). In four of the BLM knockdown clones and 14 of the RTEL1 knockdown clones, the entire 3-kbp spacer sequence was copied from the repair template into the upstream *mCherry* (Figure 5, B and C). This extensive repair synthesis might occur through multiple cycles of strand exchange, as is believed to occur in *Drosophila* gap repair by SDSA (McVey *et al.* 2004), or through a single, continuous synthesis event. Among the 17 examples in which the entire spacer was copied, one from each knockdown sample had lost *neo*, a structure that is most consistent with a dHJ being resolved to give a crossover (Figure 2D and Figure 5C). The other 15 may have arisen by long-tract SDSA or by dissolution or noncrossover resolution of a dHJ (Figure 2D and Figure 5B).

There were additional repair events from knockdown cells that also had evidence of long-tract synthesis. Three events from BLM knockdown and seven from RTEL1 knockdown had a subset of the spacer copied into the upstream *mCherry* (Figure 2E and Figure 5D). These are most likely hybrid repair that involved long-tract synthesis followed by end joining. There was one event from the BLM knockdown that had an intact *I-Sce*I site but had lost *neo* (Figure 5E). The source of this event and whether it occurred following *I-Sce*I cleavage is unknown.

### Roles of BLM and RTEL1 in SDSA

It was surprising that none of the helicase knockdowns led to decreased SDSA, since orthologs of all of these have been suggested to promote SDSA. One possibility is that there is redundancy among two or more of these proteins. This possibility can be addressed through simultaneous knockdowns or use of doubly mutant cells. In the case of BLM and RTEL1, knockdown led to dramatic increases in HDR, both in the frequency of red-fluorescing cells (Figure 3C) and in the fraction of nonfluorescing cells that had evidence for HDR (zero out of 45 for NT compared to 11 out of 34 for BLM knockdown and 24 out of 133 for RTEL1 knockdown). This is consistent with a reported increase in gene conversion in the DR-GFP assay after siRNA knockdown of BLM (Paliwal *et al.* 2014).

Molecular analysis of repair events provides some insights into sources of increased HDR. Several repair products from both BLM and RTEL1 knockdowns had extensive synthesis (Figure 5). Extensive synthesis has also been reported in another assay when the HDR proteins BRCA1 or CtIP were knocked down, and it was suggested that these proteins prevent long-tract HDR (Chandramouly *et al.* 2013). BLM and RTEL1 might prevent long-tract HDR through their D-loop disruption activities (Van Brabant *et al.* 2000; Barber *et al.* 2008). Our assay requires enough synthesis from both ends of the DSB to allow annealing within the 350-bp repeat (Figure 2B), or sequential strand invasion and synthesis (Figure 2C). If BLM and RTEL1 normally disrupt D-loops after less than a few hundred nucleotides of synthesis, the nascent strands would not be able to anneal at the 350-bp repeat and we would see reduced SDSA. In this scenario, we might expect the NT sample to have some hybrid repair events with short synthesis tracts (as in Figure 2E but with only part of the 350-bp repeat on one or both ends and none of the spacer sequence). We did not detect any such products, but it is possible our sample size was not large enough to observe these infrequent events.

Some repair products in knockdown cells had synthesis that spanned the entire 3-kbp spacer (*e.g.*, Figure 5B). These could generate dHJ intermediates that would then be resolved or dissolved. In the case of RTEL1 knockdown, only one out of 13 events in which the entire spacer was copied had the structure expected of dHJ crossover resolution (Figure 5C). If crossover and noncrossover resolution occur at equal frequencies, then either SDSA or dHJ dissolution were likely responsible for most of these events. The BTR complex, which contains the BLM helicase, is believed to be the major or sole dissolvase (Wu and Hickson 2003; Seki *et al.* 2006; Dayani *et al.* 2011). Although there was also only a single event in the BLM knockdown suggestive of dHJ crossover resolution (Figure 5C), there were only three other events in which the entire spacer was copied; it is possible that all three of these came from dHJ resolution that had a noncrossover outcome.

In our assay, SDSA may occur if both ends of the DSB engage with the template. This suggests the possibility that a function of BLM and RTEL1 is to ensure that only one end engages with the repair template. Among the 10 repair events in which only part of the spacer was copied into the upstream *mCherry* (Figure 5D), all of them appeared to have synthesis from the left end only. This is in contrast to the *Drosophila P*{*w^a^*} excision assay, where most repair events have several kilobase pairs of synthesis from both ends of the break (Adams *et al.* 2003). The disparity could arise from the difference between organisms or tissues, different structures of the DSB ends (4 nt 3′ overhangs for *I-Sce*I; 17 nt 3′ overhangs for *P* element excision), or distance between the sequence homologous to the left side of the DSB and the sequence homologous to the right side (∼3.5 kbp for this assay but >14 kbp for *P*{*w^a^*}).

The cases of partial copying of the spacer most likely derive from hybrid repair in which the initial steps of SDSA are executed but dissociation of the nascent strand does not reveal complementary sequences for annealing, so repair is instead completed by DNA polymerase Θ-mediated end joining (also called microhomology-mediated end joining) (Chan *et al.* 2010; Wyatt *et al.* 2016). As in SDSA, these D-loops must have been disassembled by another helicase than the one knocked down, or by residual helicase present after the knockdown.

Knocking down BLM did lead to elevated crossovers in the crossover assay (Figure 4D). This result was expected, based on phenotypes like elevated sister chromatid exchange (German *et al.* 1977). However, there was also an overall increase in HDR, as noncrossovers were also elevated. Thus, knocking down BLM resulted in elevated HDR in the DR-GFP assay (Paliwal *et al.* 2014), our SDSA assay, and our crossover assay. This might be expected if knockdown affects the cell cycle profile, such that more cells are in S or G2 phases and therefore more likely to repair by HDR instead of NHEJ. We conducted cell cycle profiling of cells in which BLM was knocked down, but did not detect any significant differences in the cell cycle profile compared to the NT control (Figure S4 in File S1).

The causes of increased HDR when BLM or RTEL1 is knocked down remain unknown. This is an interesting area for future investigation, as understanding this unexpected effect will no doubt reveal important functions of these proteins.

### Concluding remarks

We have demonstrated that our assays efficiently detect DSB repair by SDSA or that lead to crossovers, and that these assays can be used to study the effects of knocking down or knocking out different repair genes. Strengths of the assays include the ease of identifying the SDSA or crossover outcomes and the ability to investigate other types of repair based on structures of repair products. We did not determine whether the lines we used had only a single copy of the assay construct integrated (see *Materials and Methods*), but analyses of cells exposed to *I-Sce*I strongly argue that there was only one insertion location in both cases. In the SDSA assay, an average of 2% of cells acquired red fluorescence in any given experiment. If there were insertions at two different sites repairing independently, we would expect that in the vast majority of cases SDSA would occur in only one of the two insertions. PCR across the *I-Sce*I site would give two bands, one corresponding to the original construct and a larger band resulting from replacement of the 350-bp fragment. In opposition to this expectation, 55 out of 55 red-fluorescing clones examined had only the larger band. Similarly, crossovers in the crossover assay result in loss of mCherry. If there were several integrations every site would have to lose mCherry simultaneously to be scored as a crossover. It remains possible that one or both constructs integrated in a tandem array in the lines we used. If this happened, then it is likely that all *I-Sce*I sites were cut, leaving some extrachromosomal fragments but only a single chromosomal repair template. It is unknown what effect the extrachromosomal fragments would have on repair of the chromosomal DSB.

These assays can be modified to tailor their use in addressing specific questions. With the development of CRISPR/Cas9 genome editing (Cong *et al.* 2013; Mali *et al.* 2013), gene knockouts could be done instead of knockdowns, at least for genes that are not essential in the timeframe of these assays. It may be advantageous to use other cell lines for this approach, as U2OS cells have more than two copies of many genes (Forbes *et al.* 2015). For some questions, it would be informative to incorporate SNP markers into the template so that gene conversion tract properties could be measured at a higher resolution than reported here. Differences between the two 350-bp repeats in the SDSA assay could be used to identify cases of template switching or two-ended invasions. We did not attempt to develop high-throughput sequencing of repair products, but amplification of the entire SDSA module with single-molecule tagging would make it possible to sequence a large number of independent repair events simultaneously. Finally, various distance parameters, such as changes to the amount of synthesis required to reach the repeats (they are immediately adjacent to the DSB in our assay, but >5 kbp into the gap in the *P*{*w^a^*} assay) or length of the repeats (350 bp in this assay, 275 bp in *P*{*w^a^*}) could provide insight into the frequency of template switching, the length of a typical synthesis tract, and the ability to repair larger gaps.

Elucidating details of SDSA and crossover repair is important for understanding DSB repair in general, but will also prove vital in future optimization of CRISPR/Cas9 gene replacement or integration strategies that have been hypothesized to occur through SDSA (Byrne *et al.* 2015). We believe both assays we describe can be useful in achieving these goals.

## Supplementary Material

Supplemental material is available online at www.g3journal.org/lookup/suppl/doi:10.1534/g3.116.037390/-/DC1.
