# Supplementary material for: Human Cell Assays for Synthesis-Dependent Strand Annealing and Crossing over During Double-Strand Break Repair
Source: G3 (Bethesda). 2017 Feb 7;7(4):1191–9. doi: 10.1534/g3.116.037390 (PMC5386867; doi:10.1534/g3.116.037390)
Supplement: Supplementary file 1 [file 1191FileS1.pdf]

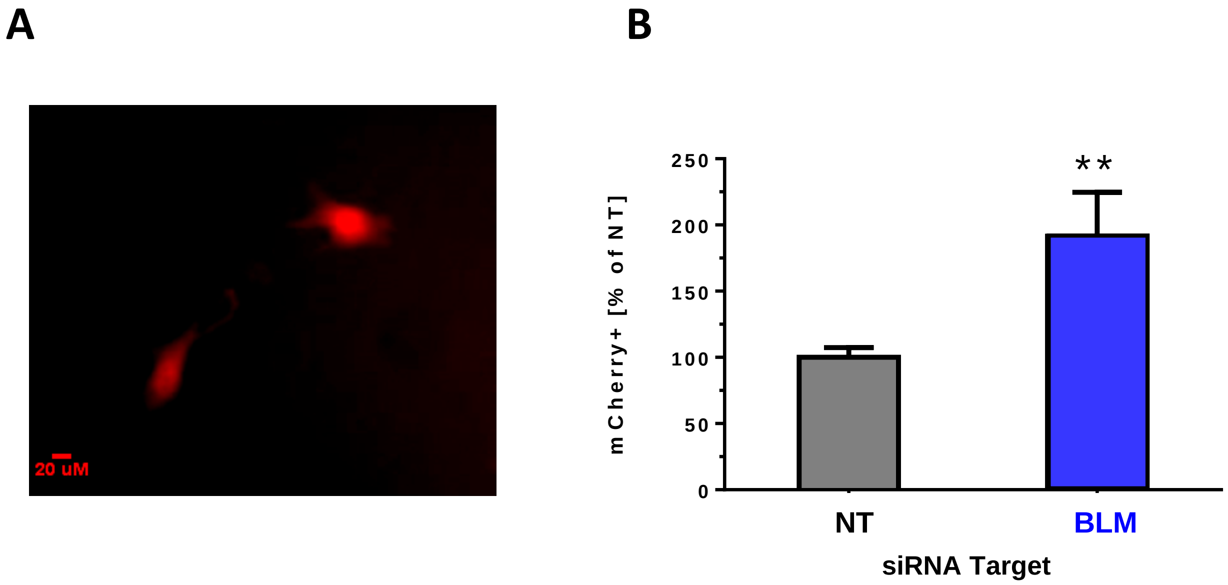

Figure S1 | SDSA assay in HeLa cells. (A) Cells exhibiting red fluorescence after infection with *I-Sce*I adenovirus. (B) Frequency of red-fluorescing cells normalized to the non-targeting (NT) siRNA is shown. NT mean mean was 0.5% of cells; siRNA BLM was 0.975% of cells. Bars are average of three biological replicates (treatments on different days). Error bars are standard deviation. ** *P* = 0.0017.

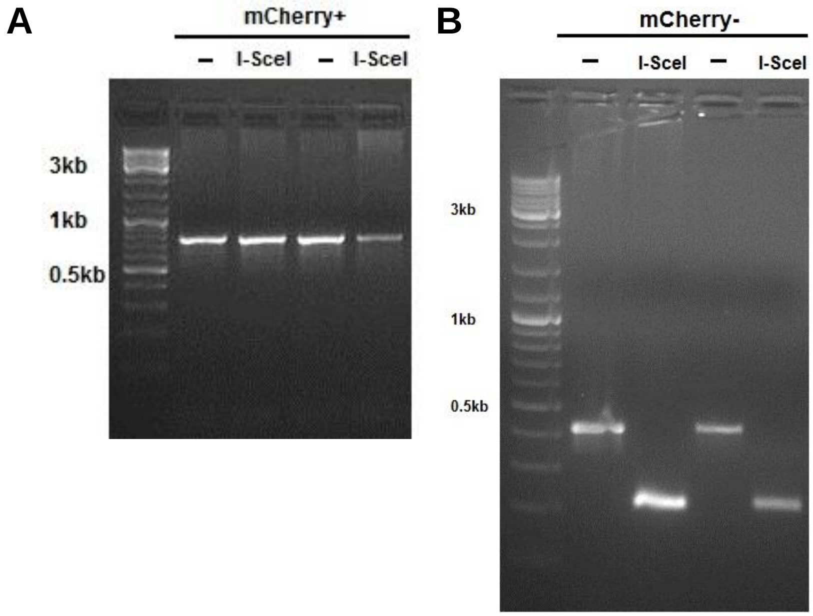

Figure S2 | Retention of *I-Sce*I site. DNA was extracted from cells treated with non-targeting siRNA before infection with *I-Sce*I adenovirus. The region surrounding the *I-Sce*I site was amplified using nehI_F and hindiii_R primers (see Table S1) and the product was either cut with *I-Sce*I or not prior to running on a 1.5% agarose gel. (A) Two cell clones that exhibited red fluorescence. Products show the size expected for SDSA (783 bp) and are not cut by *I-Sce*I. (B) Two cell clones that did not display red fluorescence. These have the same product size as the starting clone (433 bp) and the *I-Sce*I site is intact in both.

A

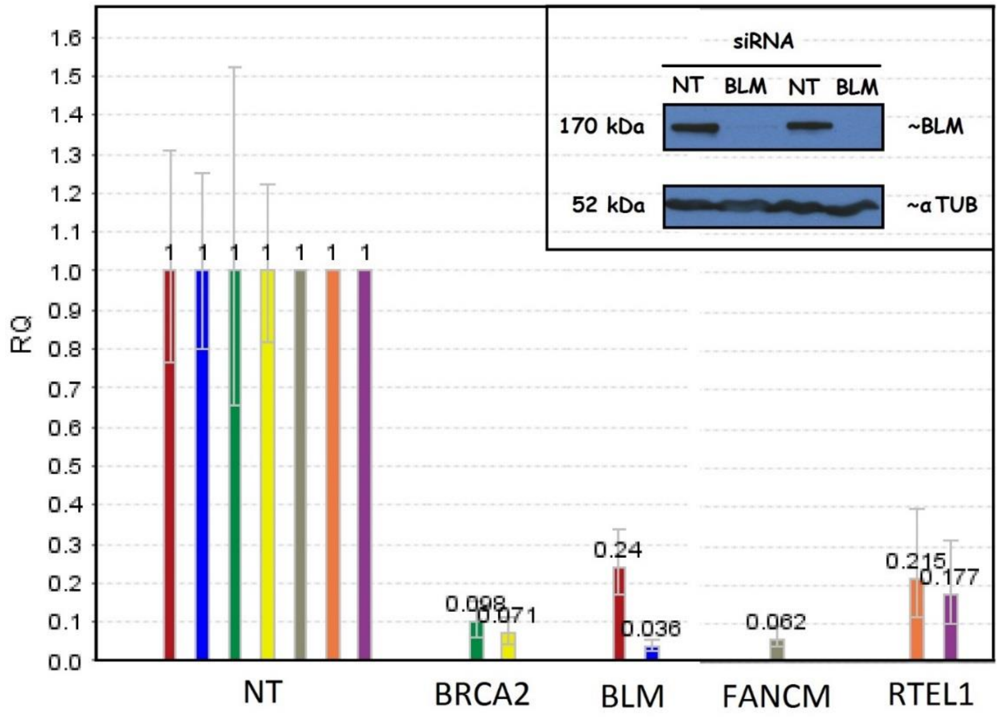

B

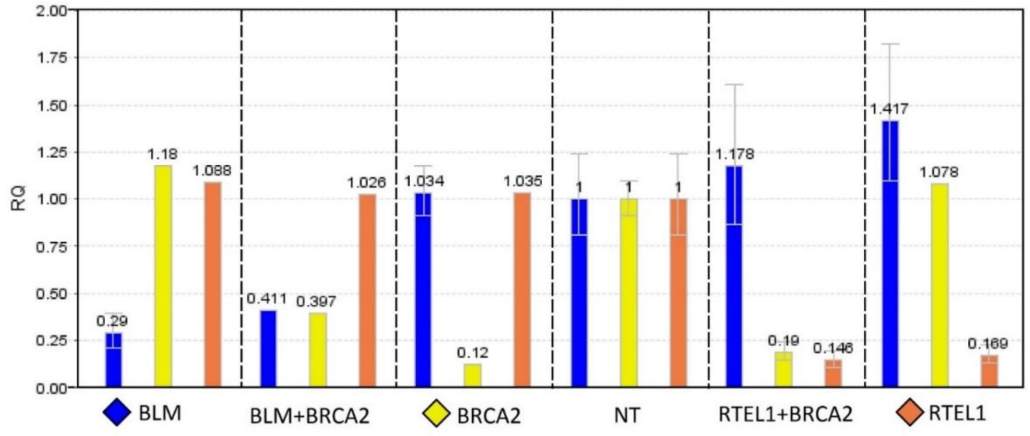

Figure S3 | Knockdown efficiency. (A) Knockdown efficiency of the cells treated with single siRNA targeting BRCA2, BLM, FANCM, RTEL1 using 1) qPCR with the primers sets depicted as different colors (green and yellow = BRCA2; blue and red = BLM; gray = FANCM; orange and purple = RTEL1) and 2) Western blot with BLM antibodies (top left). (B) Knockdown efficiency of the cells treated with double siRNAs targeting BRCA2, BLM, RTEL1 in combinations as indicated, using qPCR with the primers sets depicted as different colors (blue = BLM; yellow = BRCA2; orange = RTEL1)

Primer		Sequence	Amplicon Size (bp)
1	NheI_F	CGTGACGCTAGCGCTACCGG	433 if intact *I-Sce*I, 783 if SDSA
2	HindIII_R	CGAAGCTTGAGCTCGAGATC	
3	neo_F	GGATGAGGATCGTTTCGCATG	996
4	neo_R	CATAGAAGGCGGCGGTGGAATCG	
5	pSDSAprom_F	GGCCAAGATCTGCACACTGG	
6	SDSAseq1_R	CCTCGCAGCAAATGCTGGATC	1298
7	SDSAseq2_R	CAGAGAATCAACTGGCTGAC	1498
8	SDSAseq3_R	CGTGAGAGAAGCTCTATGGC	1707
9	SDSAseq4_R	CTGTGGGCCTATTACTCCAG	2640
10	SDSAseq5_R	GTCCCACGCGTCGACAAGGC	4112

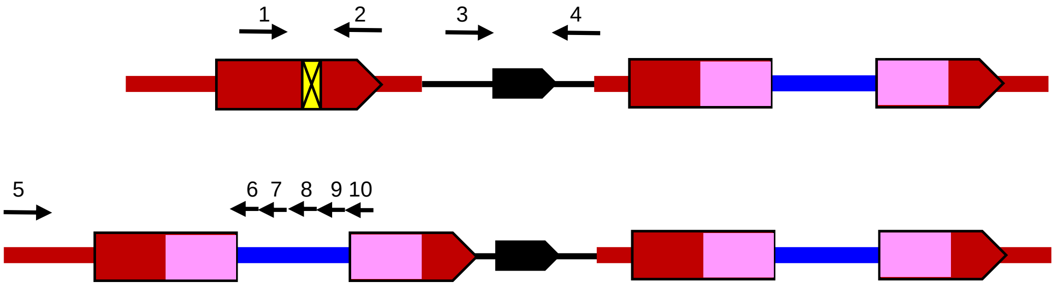

Table S1. | PCR primers used in this study and the predicted amplicon size.
Primers are grouped according to pairs used in various PCR reactions. The below shows the locations of the primers on the original construct (top) or the hybrid repair product (bottom).

Date	Well	NT	BRCA2	BLM	BLM + BRCA2	RTEL1	RTEL1 + BRCA2	FANCM	WRN	RECQL5	FBXO18
4/9/2015	1	0.7		1.9				0.6			
	mean	0.7		1.9				0.9			
	%NT	100		270				130			
5/1/2015	1	0.6		1.2				0.4			
	2	0.7		1.2				0.8			
	3	0.6		1.3				0.8			
	mean	0.6		1.2				0.7			
	%NT	100		200				120			
5/13/2015	1	2.1		2.5				2.0			
	2	2.0		2.9				1.9			
	3	2.0		2.8				2.0			
	mean	2.0		2.7				2.0			
	%NT	100		140				100			
3/6/2016	1	1.04	0.77	2.15		2.41					
	2	1.11	0.77	2.18		2.29					
	3	1.18	0.82	2.10		2.25					
	4	1.12	0.80	2.12		2.30					
	mean	1.11	0.79	2.14		2.31					
	%NT	100	71	193		208					
4/6/2016	1	2.28	1.15	2.81	1.99	3.07	1.94				
	2	2.18	1.13	2.75	2.13	3.09	1.92				
	3	2.15	1.02	2.83	2.16	2.97	1.72				
	4	2.07	1.09	2.75	2.04	3.07	1.81				
	mean	2.17	1.10	2.79	2.08	3.05	1.85				
	%NT	100	51	129	96	141	85				
9/29/2016	1	1.93	1.10	3.44	1.85	3.22	1.82				
	2	2.08	1.16	3.16	1.97	3.40	1.94				
	3	2.06	1.00	3.25	1.74	3.30	1.81				
	4	2.07	1.14	3.08	1.86	3.00	1.81				
	mean	2.04	1.10	3.23	1.86	3.23	1.85				
	%NT	100	54	159	91	159	91				
10/7/2016	1	0.79	0.45	1.28	0.56	1.46	0.52				
	2	0.92	0.48	1.30	0.56	1.28	0.50				
	3	0.82	0.54	1.30	0.55	1.52	0.55				
	4	0.83	0.47	1.29	0.63	1.40	0.56				
	mean	0.84	0.49	1.29	0.58	1.41	0.53				
	%NT	100	58	154	68	168	58				
10/6/2016	1	0.79	0.37	1.25	0.66	1.05	0.45				
	2	0.88	0.39	1.35	0.68	1.13	0.57				
	3	0.85	0.39	1.28	0.71	1.02	0.49				
	4	0.94	0.41	1.40	0.74	1.11	0.51				
	mean	0.87	0.39	1.32	0.70	1.08	0.50				
	%NT	100	45	153	81	125	58				

Table S2 | Results of siRNA knockdown on acquisition of mCherry fluorescence in SDSA assay.
See next page for continuation of table and legend.

Date	Well	NT	BRCA2	BLM	BLM + BRCA2	RTEL1	RTEL1 + BRCA2	FANCM	WRN	RECQL5	FBXO18
6/9/2015	1	1.1								1.0	1.1
	2	1.1								0.9	1.2
	mean	**1.1**								**1.0**	**1.2**
	%NT	**100**								**95**	**105**
7/2/2015	1	1.0								1.0	1.0
	2	1.0								1.0	1.0
	3	0.9								0.9	1.0
	4	1.1								0.8	0.9
	mean	**1.0**								**0.9**	**0.9**
	%NT	**100**								**93**	**98**
6/2/2016	1	3.16								3.38	
	2	3.09								3.46	
	3	3.26								3.12	
	4	3.11								3.67	
	mean	**3.15**								**3.41**	
	%NT	**100**								**108**	
6/8/2016	1	1.16		1.82					1.21		
	2	1.22		2.00					1.22		
	3	1.18		1.91					1.51		
	4	1.15		1.80					1.40		
	mean	**1.18**		**1.88**					**1.33**		
	%NT	**100**		**160**					**113**		
6/8/2016	1	1.16							1.21		
	2	1.15							1.40		
	mean	**1.18**							**1.33**		
	%NT	**100**							**113**		
6/14/2016	1	3.31							3		
	2	2.89							2.98		
	mean	**3.10**							**3.03**		
	%NT	**100**							**98**		

Table S2 | Results of siRNA knockdown on acquisition of mCherry fluorescence in SDSA assay. For each experiment (different dates), the table lists the percentage of cells expressing mCherry, based on flow cytometry. Most experiments had several technical replicates (different wells from splitting a pool of cells prior to addition of siRNA). The mean of the technical replicates (red numbers) was taken as the value for the entire experiment; these means were used in statistical comparisons of siRNA treatments. Values in blue are normalized to the within-experiment non-targeting siRNA control; these values are graphed in Figure 3C.

Date	Well	NT		BLM	
		GFP only	GFP + mCherry	GFP only	GFP + mCherry
5/28/2015	1	0.60	16.59	1.59	27.00
	2	0.61	16.43	2.11	28.57
	3	0.60	15.29	1.82	26.53
	**mean**	**0.60**	**16.10**	**1.84**	**27.37**
	**%NT**	**100**	**100**	**305**	**170**

Table S3 | Results of siRNA knockdown on acquisition of GFP fluorescence and loss of mCherry fluorescence in the crossover assay.
This experiment was done once with three technical replicates (different wells from splitting a pool of cells prior to addition of siRNA).

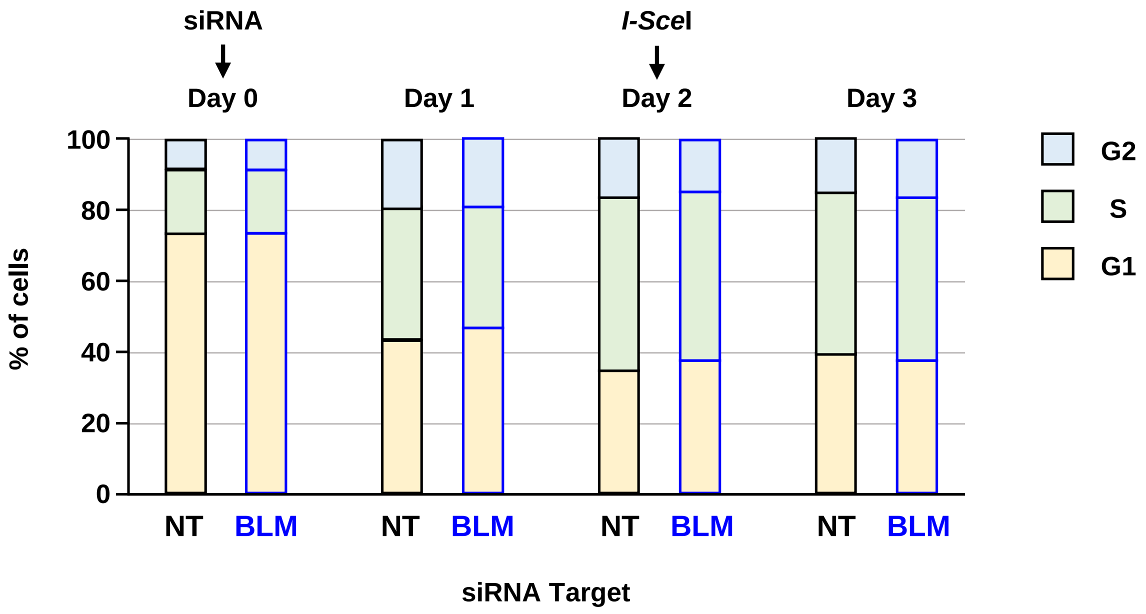

Figure S4 | Cell cycle profiling. An aliquot of U2OS-pGZ-DSB-SDSSA cells was taken prior to siRNA transfection and on each of the next three days. Cells were fixed and strained with propidium iodide according to standard protocols. Data were acquired on a BD LSRFortessa flow cytometer and analyzed using ModFit LT, version 4.1 (Verity Software House, Topsham, ME).
